# The autophagy gene product BEC-1 supports normal aging and neurodevelopment in *Caenorhabditis elegans* I

**DOI:** 10.17912/micropub.biology.000099

**Published:** 2019-06-14

**Authors:** Nicholas Ashley, Andrea M Holgado

**Affiliations:** 1 St. Edward's University, Department of Biological Sciences, Austin, TX 78704, USA

**Figure 1.**
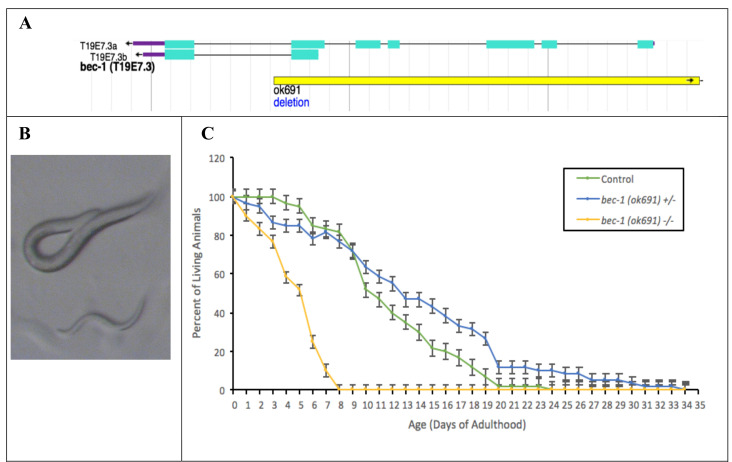
***C. elegans bec-1(ok691)* mutants have a shorter lifespan**. (A) Schematic of *bec-1* gene shown in light blue and *bec-1* (*ok691)* deletion mutation in yellow (wormbase.org). (B) Image of homozygous *bec-1* *(ok691)* mutant surviving embryonic lethality (bottom), and the heterozygous counterpart (top). (C) Lifespan of homozygous *bec-1 (ok691)* mutants, heterozygous *bec-1* *(ok691)* mutants, and control animals were monitored. Data plotted are mean ± 1 SEM, n=60. Statistical analysis was performed using a non-parametric Kruskal-Wallis (p< .001).

## Description

Macroautophagy (hereon referred as Autophagy) is a cellular housekeeping mechanism that uses a double membrane to target and engulf cell products forthe formation of autophagosomes. These double membrane organelles then fuse to lysosomes where cell products are degraded and recycled (Nakamura and Yoshimori, 2018). Reports show that autophagy plays an important role in pathogen defense, development, starvation adaptations, and aging (Mizushima et al., 2008). Identifying molecular mechanisms responsible for autophagy in mammalian cells has been possible as a result of studying model systems, such as *Saccharomyces cerevisiae*. Autophagy related genes (Atg) are evolutionarily conserved; therefore, research of autophagy in simpler organisms have informed the roles of Atg in mammalian cells (Ruck et al., 2011; Mercer et al., 2018; Tyler and Johnson, 2018). Analysis of autophagy mutants in *C. elegans*revealed that *bec-1/Atg6/Beclin 1* is essential for dauer development, a quiescent state that survives harsh conditions such as lack of nutrients, high nematode density, and high temperatures by inducing autophagy (Meléndez et al., 2003; Meléndez and Levine 2009).

To further investigate the phenotypes associated with the *bec-1(ok691)* mutation, we studied nematodes possessing a 3000 base pair deletion mutation (allele *ok691*) in the *bec-1* locus (Figure 1A). Previous reports show that the *bec-1(ok691)* mutation is lethal, however a small proportion of homozygous *bec-1(ok691)* animals reach adulthood due to maternal effect, but do not reproduce due to sterility (Figure 1B, Melendez and Levine 2009). Our analysis of survival throughout adulthood shows that lifespan is significantly reduced in homozygous *bec-1(ok691)* mutants (Figure 1C). These nematodes do not live longer than 8 days of adulthood and 50% of animals died at day 5 of adulthood. In contrast, heterozygous *bec-1(ok691)* mutant lifespan is not significantly different from control animals, ruling out previously discovered haploid insufficiency effects of Beclin 1 for autophagic activity in *C. elegans* (Sinha and Levine, 2008). This conclusion should be considered as preliminary as we have not verified by an alternative line of investigation (e.g., a second allele or transgene rescue) that the observed phenotypes are specific to *bec-1(ok691)*.

## Methods

Synchronizing:

Mixed stage nematodes grown on NGM plates at 20 °C were floated off using 1 mL of M9 reagent and collected in 1.5 mL tubes. Tubes containing animals were centrifuged at 9.3 × g for 1 minute. After centrifugation, the supernatant was discarded and the worm pellet was kept and treated with 1 mL of Alkaline Bleach (2.0% bleach (VWR), 0.5N NaOH) for 7 minutes at room temperature with occasional mixing. Once the 7-minute treatment concluded, bleached animals were centrifuged at 9. 3 × g for 2 minutes to collect eggs. Pelleted eggs were washed 3 times with 1 mL of M9 and centrifuged for 1 min. at 9.3 × g. After centrifugation, the supernatant was discarded and the pelleted eggs were suspended. Two drops of resuspended eggs were placed onto seeded NGM plates.

Lifespan:

Synchronous nematodes were placed on seeded NGM plates on day 0 of adulthood. A total of 3 replicas per group of 20 individuals were assessed each day for survival. Survival was defined as the presence of movement after nose touch and recorded daily. Living animals were transferred on a daily basis onto freshly seeded NGM plates. Lifespan was reported as percent of living animals over time.

## Reagents

Strains CZ1200 and VC517 were obtained from the *C. elegans* Genetics Center. CZ1200 contains the transgene *juIs76*[*unc25p*::GFP] which drives the expression of GFP in d-type motor neurons. Strain AMH50 was produced in our laboratory by crossing CZ1200 with VC517 *bec-1(ok691)/nT1[qIs51]*. AMH50 possess the balanced lethal mutation *bec-1(ok691)/nT1[qIs51]* and the transgene *juIs76*, {*bec-1(ok691)* IV/*nT1*[*qIs51*](IV;V);*juIs76*[*unc-25p*::GFP] II}.
